# The thymoquinone-induced production of reactive oxygen species promotes dedifferentiation through the ERK pathway and inflammation through the p38 and PI3K pathways in rabbit articular chondrocytes

**DOI:** 10.3892/ijmm.2014.2014

**Published:** 2014-11-27

**Authors:** SEON-MI YU, SONG-JA KIM

**Affiliations:** Department of Biological Sciences, Kongju National University, Gongju 314-701, Republic of Korea

**Keywords:** thymoquinone, chondrocytes, reactive oxygen species, dedifferentiation

## Abstract

Dedifferentiation and inflammation are major features of cartilage degeneration during the pathogenesis of osteoarthritis (OA). Thymoquinone (TQ) is the major compound of black seed oil isolated from *Nigella sativa* with various beneficial or harmful effects on several diseases; however, its effects on the dedifferentiation and inflammation of chondrocytes have not yet been characterized. In the present study, we investigated whether TQ regulates the dedifferentiation and inflammation of rabbit articular chondrocytes, focusing on the production of reactive oxygen species (ROS) in rabbit articular chondrocytes. TQ induced the generation of ROS in a dose-dependent manner, as shown by staining with the fluorescent probe, 2′–7′-dichlorofluorescein diacetate. We confirmed that TQ induced dedifferentiation by measuring the loss of type II collagen and the reduction in chondroitin sulfate proteoglycan levels. TQ also caused inflammation by inducing the expression of cyclooxygenase-2 (COX-2) and prostaglandin E_2_ (PGE_2_). The antioxidant, N-acetyl cysteine (NAC), prevented the dedifferentiation and inflammation which was generated by the TQ-induced production of ROS. Furthermore, TQ caused a dose-dependent increase in p38, phosphorylated extracellular signal-regulated kinase (p-ERK) and phosphoinositide 3-kinase (PI3K) expression. NAC abrogated this effect and attenuated the dedifferentiation and inflammation which was generated by the TQ-induced production of ROS. To identify the ROS-regulated pathways, we treated the chondrocytes with the p38 inhibitor, SB203580, the MEK inhibitor, PD98059, and the PI3K inhibitor, LY294002. PD98059 inhibited the TQ-induced dedifferentiation and SB203580 and LY294002 prevented the TQ-induced inflammation. These findings suggest that the TQ-induced production of ROS causes dedifferentiation through the ERK pathway and inflammation through the PI3K and p38 pathways in rabbit articular chondrocytes.

## Introduction

Osteoarthritis (OA), a degenerative joint disease, is a multifactorial process in which mechanical factors play a central role and is characterized by alterations in the structure and function of the whole joint (1). OA involves the entire joint organ, including the subchondral bone, meniscus, ligaments, periarticular muscle, capsule and synovium, and is associated with risk factors, such as age, gender, obesity, prior joint injury, genetic predisposition and mechanical factors, including malalignment and abnormal joint shape (2). During skeletal development, chondrocytes differentiate from mesenchymal progenitors to synthesize the cartilage (3). Differentiated chondrocytes express cartilage-specific collagens II, IX and XI. Under normal conditions, chondrocytes rest in a non-stimulated steady state and maintain the synthesis of proteoglycans and other non-collagen molecules (4).

Prostaglandins are produced by cyclooxygenases (COX) from arachidonic acid and are induced in arthritic joints (5). COX has three forms: COX-1, COX-2 and COX-3. Whereas COX-1 is constituvely expressed in various cell types to maintain homeostasis, COX-2 is the inducible form of COX, implicated in prostaglandin synthesis in the inflammatory response and has been associated with osteoarthritic cartilage (6). COX-3 is a recently described variant of COX-1 and is also known as COX-1 VI. However, to date, there is not much conclusive evidence available regarding the existence of COX-3 protein (7).

Reactive oxygen species (ROS), such as hydrogen peroxide (H_2_O_2_), superoxide anion (O_2_^−^). and hydroxyl radical (^•^OH) are generally believed to be harmful to cells and tissues (8). ROS are generated by a variety of endogenous and exogenous processes through several pathways and the mitochondria are the major source of intracellular ROS (9,10). ROS are destructive to DNA and proteins (11). ROS are involved in the regulation of the production of biochemical factors involved in cartilage degradation. They may cause damage to all matrix components, either directly or indirectly by reducing matrix component synthesis (12).

Thymoquinone (TQ) is the main active component of *Nigella sativa* oil, traditionally used in the Middle East (13). In this study, we investigated the effects of TQ and the regulatory mechanisms of TQ with respect to dedifferentiation and COX-2 expression in chondrocytes, including alterations in the expression of various signaling molecules in TQ-treated chondrocytes. Several signaling cascades, including those involving phosphoinositide 3-kinase (PI3K)/Akt and mitogen-activated protein kinases (MAPKs; p38, ERK) and c-Jun N-terminal kinase (JNK), regulate the dedifferentiation of chondrocytes and COX-2 expression by modulating the generation of ROS (14,15). Although other investigators have suggested that ROS inhibit differentiation and induce COX-2 expression, the mechanisms involved have not been fully elucidated. In the present study, we investigated the molecular mechanisms through which the TQ-induced generation of ROS affects dedifferentiation and COX-2 expression in rabbit articular chondrocytes. Our results suggest that TQ induces the generation of ROS, which modulates the PI3K/Akt or MAPK signaling cascades, leading to dedifferentiation and inflammation in rabbit chondrocytes.

## Materials and methods

### Primary culture of rabbit articular chondrocytes

Articular chondrocytes were isolated from cartilage slices of 2-week-old New Zealand white rabbits (Koatech, Pyeongtaek, Korea), as previously described (16). Briefly, the cartilage slices were enzymatically dissociated in 0.2% collagenase in Dulbecco’s modified Eagle’s medium (DMEM; Gibco, Carlsbad, CA, USA). Individual cells were cultured in DMEM supplemented with 10% (v/v) fetal bovine calf serum (Gibco). The chondrocytes were grown at 37°C in the DMEM in a humidified incubator containing 5% CO_2_. Primary chondrocyte cultures at 3.5 days were treated with 0.1 % DMSO (vehicle control) or with various pharmacological reagents, including TQ (Sigma-Aldrich, St. Louis, MO, USA). The cells were treated with various inhibitors [N-acetyl-L-cysteine (NAC), 4,4′-diiso-thiocyano-2,2′-stilbenedisulphonic acid (DIDS), SP600125, SB203580, PD98059 and LY294002] for 1 h prior to treatment with TQ. NAC and DIDS were purchased from Sigma-Aldrich, and SP600125 was obtained from Biomol (Plymouth Meeting, PA, USA). The other chemicals used, SB203580 and PD98059, were purchased from Calbiochem (San Diego, CA, USA). LY294002 was obtained from Tocris Bioscience (Bristol, Avon, UK). The study was approved by the Ethics Committee of Kongju National University, Gongju, Korea.

### Western blot analysis

The cells were lysed in radioimmuno-precipitation (RIPA) lysis buffer containing protease inhibitors [10 g/ml leupeptin, 10 g/ml pepstatin A, 10 g/ml aprotinin and 1 mM 4-(2-aminoethyl)benzenesulfonyl fluoride] and phosphatase inhibitors (1 mM NaF and 1 mM Na_3_VO_4_). Protease inhibitors and phosphatase inhibitors were obtained from Sigma-Aldrich. Equal amounts of protein were mixed with electrophoresis sample buffer (Bio-Rad Laboratories, Hercules, CA, USA) and boiled for 5 min before loading onto SDS-PAGE gels. Proteins were fractionated by SDS-PAGE and transferred onto nitrocellulose membranes (Millipore, Billerica, MA, USA). The membranes were incubated with primary antibodies followed by horseradish peroxidase-conjugated secondary antibodies (Sigma-Aldrich). Primary antibodies were specific to phosphorylated (p-)p38 (#9211; Cell Signaling Technology, Beverly, MA, USA), p-ERK-1/2 (#9101; Cell Signaling Technology), p-JNK (#9251; Cell Signaling Technology), p-Akt (#9271; Cell Signaling Technology), type II collagen (MAB8887; Santa Cruz Biotechnology, Santa Cruz, CA, USA), actin (sc-1615; Santa Cruz Biotechnology) and COX-2 (#160106; Cayman Chemical Co., Ann Arbor, MI, USA). Proteins were visualized with ECL Plus reagent (Amersham Biosciences) on a Chemilumino analyzer LAS 4000 mini (Fujifilm, Tokyo, Japan).

### Chondrocyte differentiation

The chondrocytes were identified by staining for sulfate proteoglycan with Alcian blue as previously described (17). The cells were washed twice with cold PBS, fixed with 95% methanol for 2 min (−20°C) and stained overnight with 0.1% Alcian Blue 8GX (Wako Pure Chemical Industries Ltd., Osaka, Japan) in 0.1 M HCl. After washing 3 times with distilled water, the stain was extracted with 800 *μ*l of 6 M guanidine-HCl for 6 h at room temperature; optical density was measured at 595 nm.

### Measurement of ROS production

The fluorogenic marker, 7′-dichlorodihydrofluorescein diacetate (DCFH-DA; Sigma-Aldrich), was used to monitor the production of intracellular ROS. Following treatment with various concentrations of TQ for 2 h, the cells were washed twice with PBS and loaded for 30 min with DCFH-DA (10 *μ*M; Sigma-Aldrich) in DMEM without phenol red. The acetoxymethyl group on DCFH-DA is cleaved by non-specific esterases within the cell, producing a non-fluorescent charged molecule that does not cross the cell membrane. Intracellular ROS irreversibly oxidizes DCFH-DA to dichlorofluorescein (DCF), which is a fluorescent product. Following treatment, the medium was removed, the chondrocytes were collected by centrifugation and fluorescence was measured on an Flx8000 fluorometer (excitation, 485 nm/emission, 525 nm; Bio-Tek Instruments, Winooski, VT, USA). For ROS visualization by fluorescence microscopy, the cells were labeled for 30 min at 37°C in the dark with DCFH-DA (10 *μ*M) probe. The chondrocytes were washed twice with PBS. Fluorescence was observed under an inverted Olympus BX50 microscope (Olympus, Tokyo, Japan). DCF fluorescence intensity was quantified using ImageJ software (Vector Laboratories, Burlingame, CA, USA).

### Immunofluorescence (IF) staining

The chondrocytes cultured on glass coverslips were fixed in 4% paraformaldehyde at 4°C for 10 min and permeabilized with 0.1% Tween-20 in PBS for 15 min. For immunostaining, goat polyclonal antibody to type II collagen (MAB8887; 1:50 dilution; Santa Cruz Biotechnology) and anti-rabbit polyclonal antibody to COX-2 (#160112; 1:50 dilution; Cayman Chemical Co.) were used as primary and secondary antibodies, respectively. Counterstaining with DAPI (Molecular Probe) enabled nuclear visualization. Images of the cultured chondrocytes were acquired using a fluorescence microscope (Olympus BX50; Olympus).

### Measurement of prostaglandin E_2_ (PGE_2_) levels

The chondrocytes were seeded in standard 96-well microtiter plates at 1×10^4^ cells/well. Following treatment, COX-2 activity was determined by measuring PGE_2_ levels in the culture medium. PGE_2_ concentrations were determined using a standardized enzyme immunoassay (EIA) according to the manufacturer's instructions (Assay Designs, Ann Arbor, MI, USA).

### Statistical analysis

Data are expressed as the means ± SEM and analyzed by one-way analysis of variance (ANOVA). Comparisons between groups were performed by ANOVA followed by Turkey’s multiple comparison, comparing all groups to the DMSO-treated group (control). Graphs were generated using Microsoft Excel 2007. P-values <0.05 were considered to indicate statistically significant differences.

## Results

**Figure 1:**
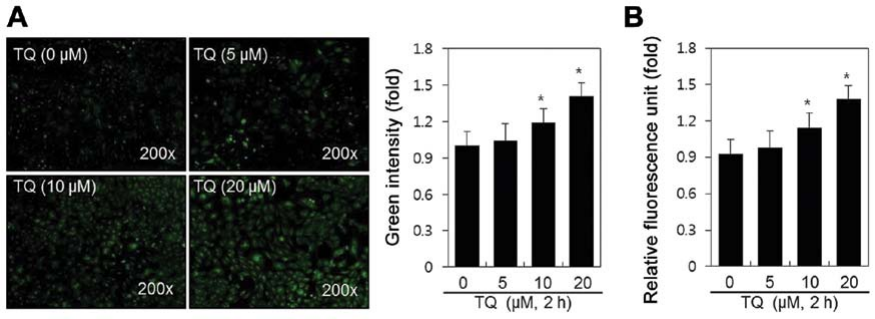
Thymoquinone (TQ) induces the generation of intracellular reactive oxygen species (ROS) in primary rabbit articular chondrocytes. (A) Chondrocytes were exposed to TQ for 2 h. Dichlorofluorescein (DCF) fluorescence intensity was observed under an inverted fluorescence microscopy (left panel). DCF fluorescence intensity was quantified using ImageJ software (right panel). (B) Chondrocytes were exposed to TQ for 2 h. ROS fluorescence was measured using an Flx8000 Bio-Tek fluorometer (B). Data are presented as the means ± SD from 3 independent experiments performed in triplicate. ^*^P<0.05, compared with the control group.

To the best of our knowledge, this is the first study assessing the effects of TQ on normal rabbit articular chondrocytes. Chondrocytes were treated with TQ (0, 5, 10 and 20 *μ*M) for 2 h, after which we observed a marked induction of ROS generation by fluorescence microscopy (Fig. 1A) and fluorometry (Fig. 1B). The dose-dependent increase in the production of ROS increased 1.4-fold after 2 h of treatment with TQ, as shown in Fig. 1B. These results indicate that TQ induces ROS production in rabbit articular chondrocytes.

**Figure 2:**
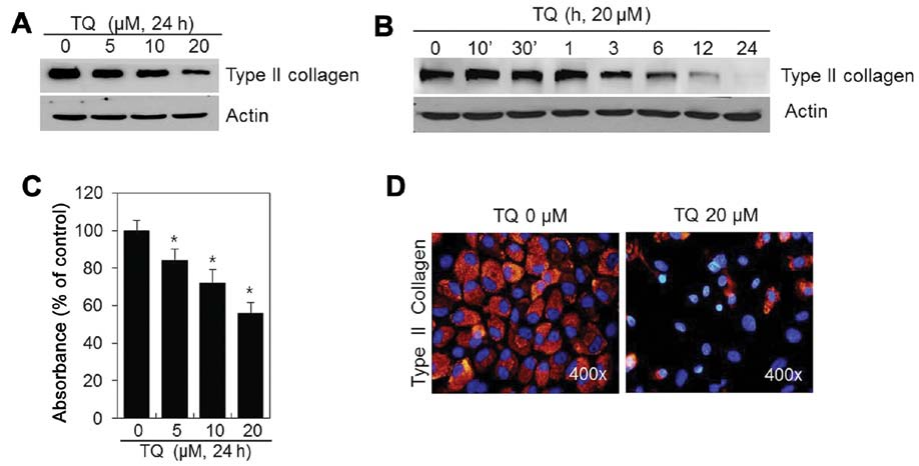
Thymoquinone (TQ) induces dedifferentiation in rabbit articular chondrocytes. (A) Chondrocytes were exposed to TQ for 24 h. (B) Cells were exposed to 20 *μ*M TQ for 24 h. (A and B) The expression of type II collagen and actin was determined by western blot analysis with actin as a loading control. Chondrocytes were treated with TQ for 24 h. (C) The synthesis of sulfate proteoglycan was determined by Alcian blue staining. Articular chondrocytes were exposed to 20 *μ*M TQ for 24 h. (D) The expression of type II collagen was determined by IF staining. Data are presented as the means ± SD from 3 independent experiments performed in triplicate. ^*^P<0.05, compared with the control group.

To determine whether TQ influences the chondrocyte phenotype, the cells were treated with various concentrations of TQ for 24 h or with 20 *μ*M of TQ for various periods of time (Fig. 2). The production of type II collagen, a differentiation marker, was inhibited in a dose- and time-dependent manner (Fig. 2A and B) following treatment with TQ. Thus, TQ is capable of inducing the dedifferentiation of chondrocytes.

We also examined the effects of TQ on the production of chondroitin sulfate proteoglycan, which accumulates during chondrocyte differentiation. The TQ-treated chondrocytes exhibited a dose-dependent decrease in sulfate proteoglycan staining in comparison to the controls (Fig. 2C). IF staining revealed that type II collagen was distributed throughout the extracellular matrix of the control cells. However, the type II collagen levels were decreased in the TQ-treated chondrocytes (Fig. 2D). These findings suggest that TQ induces the dedifferentiation of rabbit articular chondrocytes.

**Figure 3:**
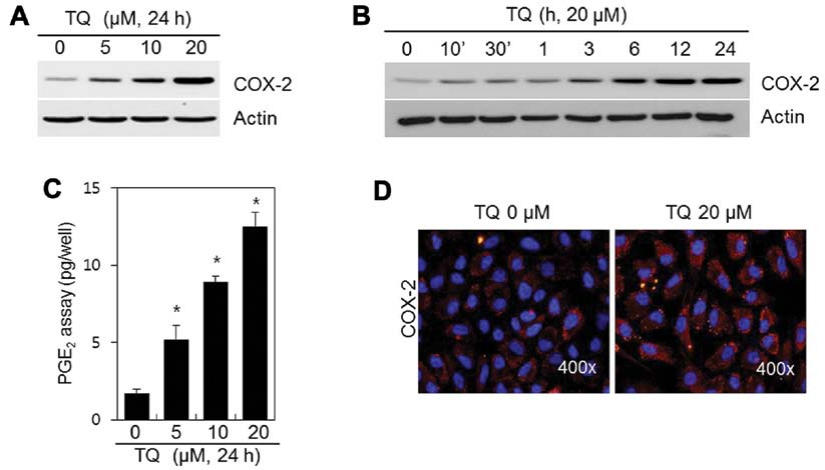
Thymoquinone (TQ) increases cyclooxygenase-2 (COX-2) expression in rabbit articular chondrocytes. (A) Primary chondrocytes were exposed to TQ for 24 h. (B) Cells were exposed to 20 *μ*M TQ for 24 h. (A and B) The expression of COX-2 and actin was determined by western blot analysis with actin as a loading control. (C) Rabbit chondrocytes were treated with TQ for 24 h. (C) Production of prostaglandin E_2_ (PGE_2_) was determined by PGE_2_ assay. (D) Chondrocytes were exposed to 20 *μ*M TQ for 24 h. (D) The expression of COX-2 was determined by immunofluorescence staining. Data are presented as the means ± SD from 3 independent experiments performed in triplicate. ^*^P<0.05, compared with the control group.

We sought to determine whether TQ affects COX-2 expression. The chondrocytes were treated with various concentrations of TQ for 24 h or with 20 *μ*M of TQ for various periods of time (Fig. 3). The expression of the inflammatory mediator, COX-2, was induced in a dose- and time-dependent manner (Fig. 3A and B) following treatment with TQ. We also found that TQ induced a marked dose-dependent induction in PGE_2_ synthesis, which is known to mediate inflammation (Fig. 3C). IF staining revealed that COX-2 was distributed in low amounts throughout the cytosol of the control cells; however, COX-2 expression was greater in the TQ-treated chondrocytes (Fig. 3D). These data suggest that TQ induces COX-2 expression in rabbit articular chondrocytes.

After observing the prominent effects of TQ on dedifferentiation and COX-2 expression in chondrocytes, we sought to elucidate the mechanisms responsible for these effects. The effects of TQ on dedifferentiation and COX-2 expression in chondrocytes were determined after 24 h of treatment with 20 *μ*M TQ. ROS play a key role in dedifferentiation and inflammation (9,18). Thus, ROS function as critical signaling molecules in various cell types, including chondrocytes.

**Figure 4:**
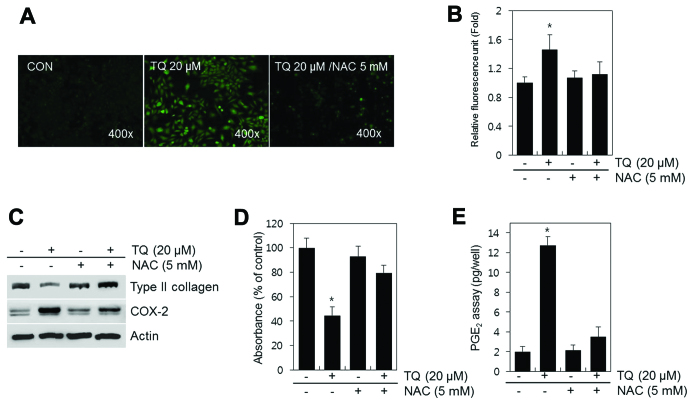
Thymoquinone (TQ)-induced dedifferentiation and cyclooxygenase-2 (COX-2) expression are blocked by an inhibitor of reactive oxygen species (ROS), N-acetyl cysteine (NAC). Chondrocytes were exposed to 20 *μ*M TQ in the absence or presence of 5 mM NAC (A and B) for 2 h or (C and D) for 24 h. ROS production was determined by fluorescence microscopy (A; magnification, ×200). (B) Reactive oxygen species (ROS) fluorescence was measured using an Flx8000 fluorometer. (C) The expression of type II collagen and COX-2 was determined by western blot analysis with actin as a loading control. (D) The synthesis of sulfate proteoglycan was detected by Alcian blue staining. (E) The secretion of prostaglandin E_2_ (PGE_2_) was analyzed by PGE_2_ assay. Data are presented as the means ± SD from 3 independent experiments performed in triplicate. ^*^P<0.01, compared with the control group.

TQ induced the production of ROS (Fig. 4A); thus, this production of ROS may mediate the TQ-induced dedifferentiation and expression of COX-2 in chondrocytes. We examined this hypothesis by assessing the effects of TQ in the presence of NAC, a ROS scavenger (Fig. 4). The TQ-treated chondrocytes were incubated with NAC (5 mM) for 24 h and then analyzed by fluorescence microscopy (Fig. 4A). In the TQ-treated cells, a 1.5-fold increase in ROS production was observed; no change was observed in ROS production in the control cells. Treatment of the chondrocytes with TQ in the presence of NAC resulted in only a 1.1-fold increase in ROS production (Fig. 4B). Blocking the generation of ROS with NAC nearly abolished the TQ-induced loss of type II collagen, as well as the increase in COX-2 expression and PGE_2_ production in chondrocytes (Fig. 4C–E).

**Figure 5:**
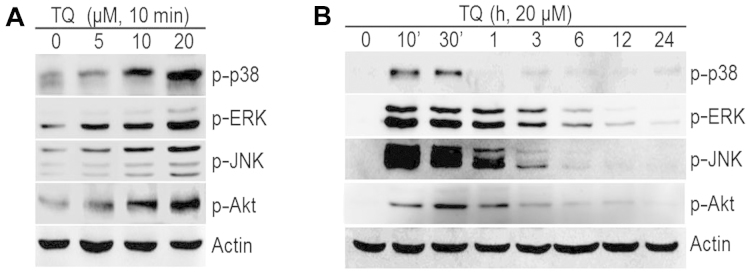
Thymoquinone (TQ) activates the PI3K/Akt and the MAPK (p38, ERK and JNK) pathways in chondrocytes. (A) Rabbit chondrocytes were exposed to TQ for 24 h. (B) Articular chondrocytes were exposed to 20 *μ*M TQ for the indicated periods of time. (A and B) The activation of p38, ERK, JNK and Akt was determined by western blot analysis with actin as a loading control. Data are presented as the means ± SD from 3 independent experiments performed in triplicate.

**Figure 6:**
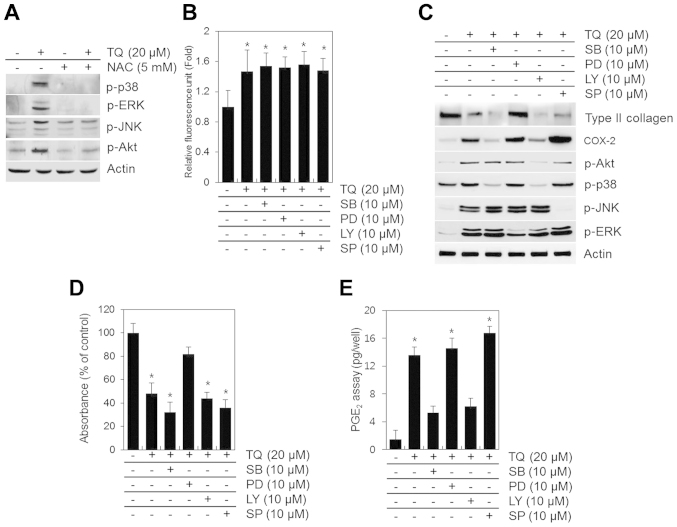
Thymoquinone (TQ)-induced reactive oxygen species (ROS) generation regulates dedifferentiation through p38 and cyclooxygenase-2 (COX-2) expression through PI3K/Akt and ERK. (A) Chondrocytes were exposed to 20 *μ*M TQ in the absence or presence of 5 mM N-acetyl cysteine (NAC) for 24 h. (A) The expression of p-p38, p-ERK, p-JNK, p-Akt and actin was determined by western blot analysis with actin as a loading control. Primary chondrocytes were exposed to 20 *μ*M TQ in the absence or presence of SB203580 (SB, PI3K inhibitor), PD98059 (PD, p38 inhibitor), LY294002 (LY, ERK inhibitor) or SP600125 (SP, JNK inhibitor) (B) for 2 h or (C–E) for 24 h. (B) ROS fluorescence was measured using an Flx 8000 fluorometer. (C) The expression of type II collagen, COX-2, p-Akt, p-p38, p-JNK and p-ERK was determined by western blot analysis with actin as a loading control. (D) The production of sulfate proteoglycan was determined by Alcian blue staining. (E) The synthesis of prostaglandin E_2_ (PGE_2_) was analyzed by PGE_2_ assay. Data are presented as the means ± SD from 3 independent experiments performed in triplicate. ^*^P<0.01, compared with the control group.

In order to gain further insight into the molecular mechanisms underlying the induction of differentiation and COX-2 expression in chondrocytes, we investigated the activation of the MAPK and PI3K pathways (Fig. 5). Our results revealed that TQ induced a dose-dependent increase in the expression of the MAPKs, p-p38, p-ERK, p-JNK and PI3K/pAkt (Fig. 5A). The TQ-induced phosphorylation of MAPKs and PI3K was long-lasting and reached maximum levels after 10 min of treatment for p-p38, p-ERK and p-JNK and 30 min for p-Akt; the levels decreased thereafter (Fig. 5B). We then determined whether the TQ-induced activation of MAPKs and PI3K is blocked by NAC (Fig. 6A). NAC inhibited the TQ-induced phosphorylation of MAPKs and PI3K (Fig. 6A). To determine the association between the TQ-induced generation of ROS, dedifferentiation and COX-2 expression and the activation of MAPKs and PI3K, we inhibited the phosphorylation of MAPKs and PI3K using specific inhibitors (SB203580 for p38, PD98059 for ERK, LY294002 for PI3K/Akt and SP600125 for JNK) prior to treatment with TQ (Fig. 6). None of these inhibitors blocked the TQ-induced generation of ROS, but some slightly inhibited the TQ-induced dedifferentiation and the expression of COX-2 (Fig. 6B and C). The inhibition of ERK by PD98059 attenuated the TQ-induced loss in type II collagen expression and proteoglycan synthesis (Fig. 6B and C). The inhibition of p38 with SB203580 or PI3K/Akt with LY294002 blocked the TQ-induced expression of COX-2 and PGE_2_ synthesis (Fig. 6C and E).

**Figure 7:**
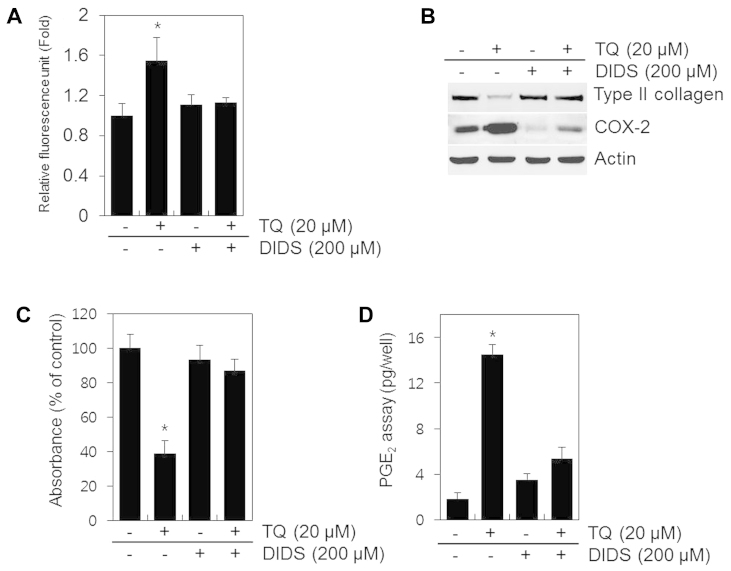
4,4′-Diisothiocyano-2,2′-stilbenedisulphonic acid (DIDS) inhibits dedifferentiation and cyclooxygenase-2 (COX-2) expression which is generated by the thymoquinone (TQ)-induced production of reactive oxygen species (ROS). Chondrocytes were exposed to 20 *μ*M TQ in the presence or absence of 200 *μ*M DIDS for (A) 2 h or (B–D) 24 h. (A) ROS fluorescence was measured using an Flx8000 fluorometer. (B) The expression of type II collagen and COX-2 was determined by western blot analysis with actin as a loading control. (C) The production of sulfate proteoglycan was determined by Alcian blue staining. (E) The synthesis of prostaglandin E_2_ (PGE_2_) was analyzed by PGE_2_ assay. Data are presented as the means ± SD from 3 independent experiments performed in triplicate. ^*^P<0.05, compared with the control group.

A recent study demonstrated that DIDS, a selective inhibitor of mitochondrial electron transport, prevents the production of ROS (19). In the present study, the cells treated with DIDS no longer produced ROS in response to TQ (Fig. 7A). Treatment with DIDS restored type II collagen expression and sulfate proteoglycan synthesis and decreased the expression of COX-2 and PGE_2_ production in the TQ-treated chondrocytes (Fig. 7B–D). These findings suggest that the inhibition of ROS production from the mitochondria by DIDS inhibits dedifferentiation and COX-2 expression and that ROS is the key source of cartilage destruction (Fig. 7).

**Figure 8:**
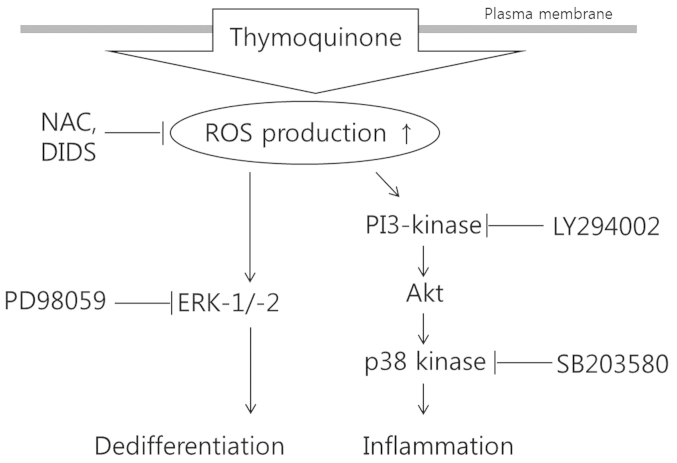
Schematic diagram of thymoquinone (TQ)-induced dedifferentiation and cyclooxygenase-2 (COX-2) expression. The TQ-induced production of reactive oxygen species (ROS) regulates dedifferentiation through the ERK pathway and modulates COX-2 expression through the PI3K and p38 pathways. These effects are blocked by treatment with N-acetyl cysteine (NAC) and 4,4′-diisothiocyano-2,2′-stilbenedisulphonic acid (DIDS).

A schematic diagram displaying the cascade of TQ -induced dedifferentiation and inflammation and the mechanisms involved is presented in Fig. 8.

## Discussion

The pathogenesis of OA is associated with risk factors, such as oxidative stress and free radicals (20,21). Oxidative stress is caused by abnormal cell metabolism exceeding the physiologicalbuffering capacity. Oxidative stress has been described to increase cellular aging, thus weakening organ function (22). Previous studies have demonstrated that OA cartilage has high oxidative activity (23,24). ROS overproduction in cartilage originates in the mitochondria and results in chondrocyte destruction, which in turn causes OA (25). ROS serve as second messengers that mediate gene transcription, cell proliferation, necrosis, apoptosis and differentiation in a variety of cell types (26). In this study, we found that TQ induced intracellular ROS production and induced dedifferentiation and COX-2 expression in chondrocytes. TQ induced a dose-dependent increase in ROS production (Fig. 1). TQ also induced the loss of type II collagen and an increase in COX-2 expression (Figs. 1 and 2), while NAC inhibited the TQ-induced dedifferentiation and inflammation (Fig. 3). These findings demonstrate that TQ is an effective inducer of ROS generation in chondrocytes, indicating that TQ may play a role in the process of cartilage destruction through ROS-mediated pathways. Since treatment of the chondrocytes with low concentrations of TQ (<5 *μ*M) than those used in the present study had no effect on ROS accumulation, type II collagen and COX-2 expression (data not shown), we used concentrations of 5–20 *μ*M in this study. In our previous study, we demonstrated that the treatment of chondrocytes with TQ (5–20 *μ*M) resulted in apoptosis, suggesting that TQ may be effectively used to elucidate the pathways or mechanisms responsible for apoptosis in chondrocytes (27). TQ may thus be a suitable reagent for determining the mechanisms responsible for dedifferentiation and inflammation.

Increasing evidence has attributed cellular damage in a variety of disorders in humans to oxidative stress that leads to ROS production, and these effects are mediated by the interaction with matrix metalloproteinases (MMPs) (28,29). Therefore, in this study, we investigated whether ROS leads to the destruction of matrix components, such as type II collagen, by activating MMPs. However, our results indicated that TQ did not affect MMP production (data not shown).

Our findings also suggested that TQ increased the expression and production of the pro-inflammatory mediators, COX-2 and PGE_2_ (Fig. 3). Dedifferentiation and inflammation are supported by an intracellular signaling network involving the PI3K/Akt and MAPKs pathways (30). Phosphorated Akt translocates to the nucleus and phosphorylates numerous target molecules to mediate signals (31). MAPKs are a family of proteins promoting a phosphorylative signaling cascade, leading to the activation of transcription factors involved either in cellular dedifferentiation and inflammation (32). It has also been reported that ROS induces dedifferentiation, inflammation and proliferation in a variety of cell types through the temporal activation of the PI3K and MAPKs pathways (31,32). In addition, several studies have linked dedifferentiation and COX-2 expression with MAPKs, p38, ERK-1/2 and JNK and PI3K/Akt. (21,33,34).

In the present study, TQ induced the activation of MAPKs and PI3K (Fig. 5) and the inhibition of TQ-induced dedifferentiation by PD98059 was due to the inhibition of ERK activation (Fig. 6C). The inhibition of p38 and PI3K decreased the TQ-induced expression of COX-2, but did not influence dedifferentiation (Fig. 6C).

As demonstrated in our study, DIDS inhibits anion channels in the mitochondrial inner membrane, thus, inhibiting ROS release from the organelle. Pre-treatment of the TQ-treated cells with DIDS abolished dedifferentiation and COX-2 expression, suggesting that the transition of ROS through anion channels may be required for the activation of the MAPK and PI3K pathways (Fig. 7). Thus, our results indicate that the TQ-induced production of ROS triggers dedifferentiation through ERK and COX-2 expression through the p38 and PI3K pathways.
